# Supplementary material for: DMRforPairs: identifying Differentially Methylated Regions between unique samples using array based methylation profiles
Source: BMC Bioinformatics. 2014 May 15;15:141. doi: 10.1186/1471-2105-15-141 (PMC4046028; doi:10.1186/1471-2105-15-141)
Supplement: Additional file 2 — DMRforPairs output for the comparison of A431-MCF7 and NA17018-NA17105. Please start from the HTML files in each folder. Available via the BMC Bioinformatics website. [file 1471-2105-15-141-S2.zip › 1394847754114233_MOESM2_ESM/A431_MCF7/figures/13785.pdf]

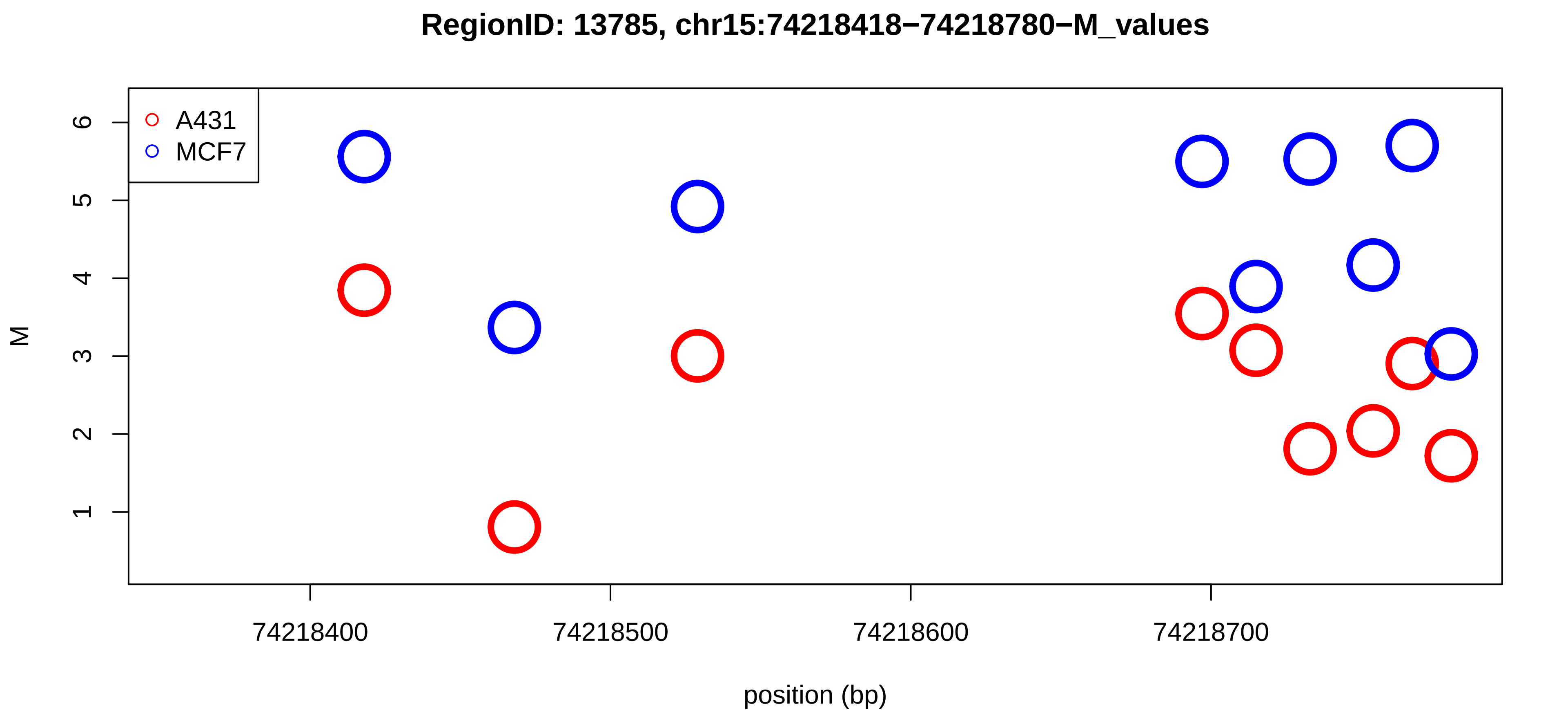

RegionID: 13785, chr15:74218418−74218780−M_values
A431
MCF7
M
1
2
3
4
5
6
74218400
74218500
74218600
74218700
position (bp)

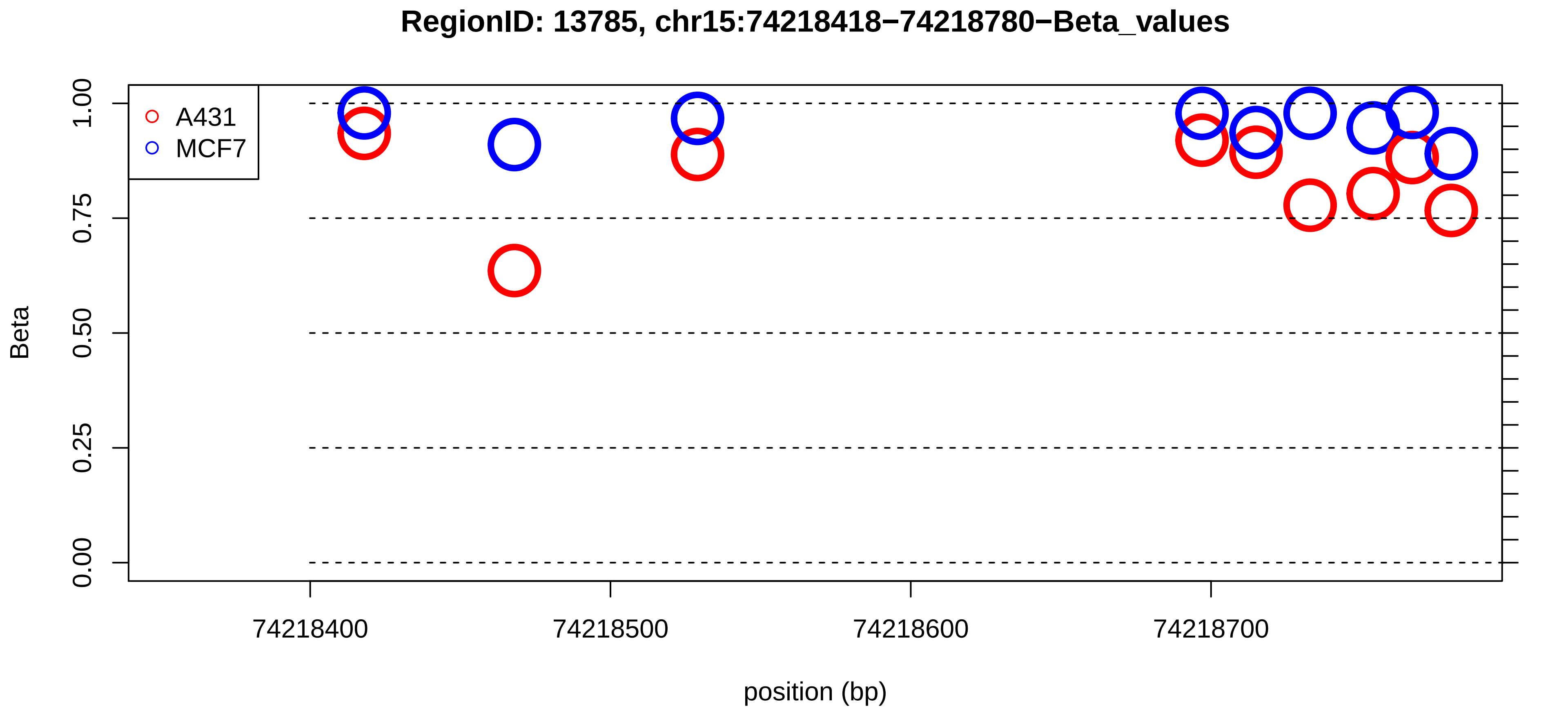

RegionID: 13785, chr15:74218418−74218780−Beta_values
A431
MCF7
Beta
0.00
0.25
0.50
0.75
1.00
74218400
74218500
74218600
74218700
position (bp)

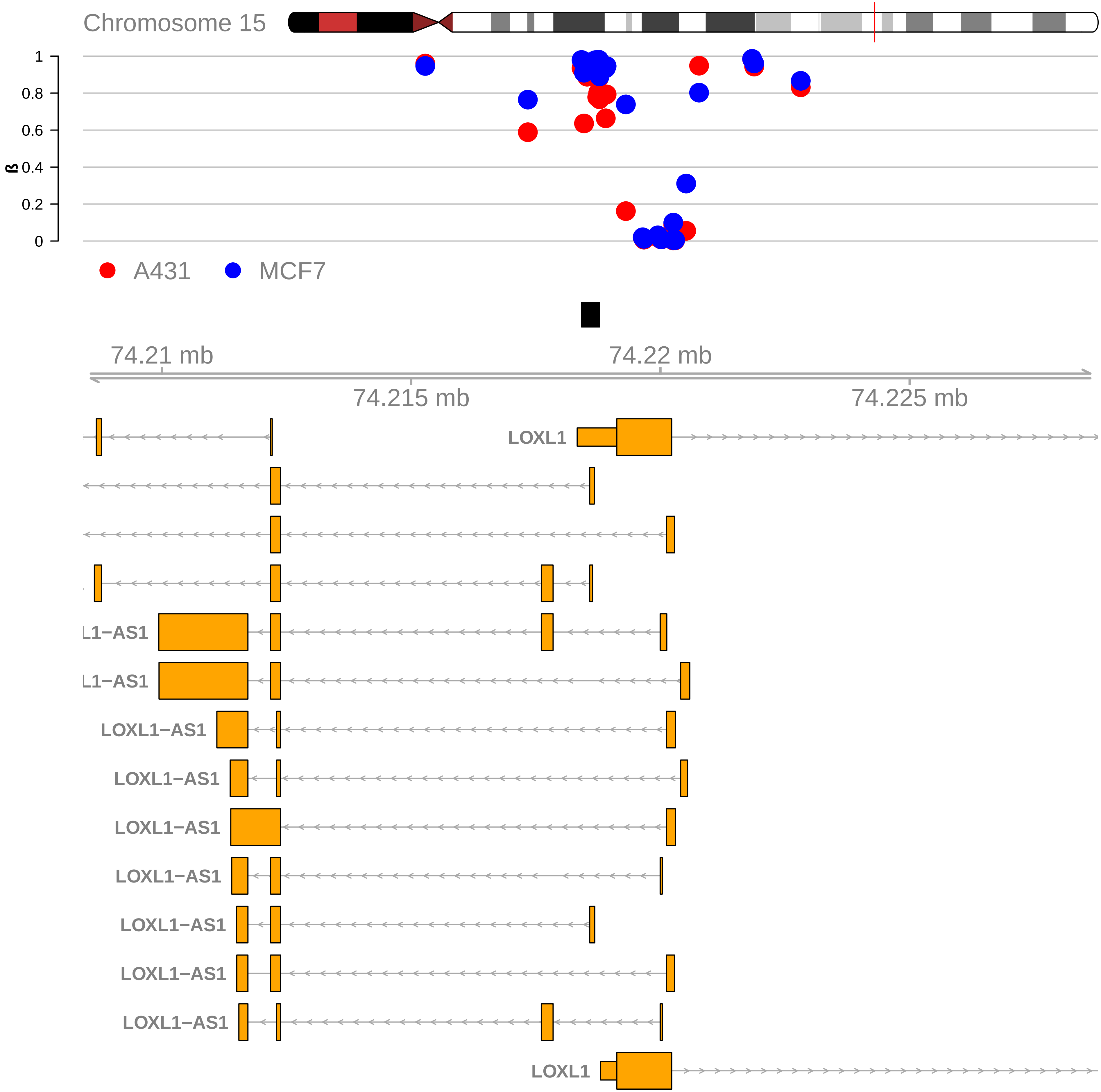

Chromosome 15
1
0.8
0.6
β 0.4
0.2
0
A431
MCF7
74.21 mb
74.22 mb
74.215 mb
74.225 mb
LOXL1
L1−AS1
L1−AS1
LOXL1−AS1
LOXL1−AS1
LOXL1−AS1
LOXL1−AS1
LOXL1−AS1
LOXL1−AS1
LOXL1−AS1
LOXL1
